# Smokers' Neurological Responses to Novel and Repeated Health Warning Labels (HWLs) From Cigarette Packages

**DOI:** 10.3389/fpsyt.2018.00319

**Published:** 2018-07-18

**Authors:** Johann F. Fridriksson, Chris Rorden, Roger D. Newman-Norlund, Brett Froeliger, James F. Thrasher

**Affiliations:** ^1^Department of Psychology, University of South Carolina, Columbia, SC, United States; ^2^Neurology, Medical University of South Carolina, Charleston, SC, United States; ^3^Department Health Promotion, Education and Behavior, University of South Carolina, Columbia, SC, United States

**Keywords:** fMRI, brain, smoking, tobacco, neural response, pictorial health warning label

## Abstract

Graphic health warning labels (HWLs) depicting bodily injury due to smoking are effective for producing changes in affect, cognition and smoking behavior in adult smokers. However, little is known about the effects of repeated presentation of graphic HWL's on the aforementioned processes. The goal of this study was to examine neural and behavioral responses to graphic HWL's and evaluate whether the repeated presentation of graphic HWL's leads to repetition suppression (RS). Smokers (*N* = 16) performed an event-related HWL cue task while blood oxygen level dependent (BOLD) signal was collected during a functional magnetic resonance imaging (fMRI) experimental session. Consistent with prior literature, graphic HWL's, as compared to scrambled images, elicited increased BOLD response in brain regions involved in self-referential and emotion processing. Importantly, BOLD response at sites in this network diminished during repeated presentation of the same HWL. These findings suggest that while novel graphic HWL's may have a significant effect on smokers' brain activity, repeated presentation may lead to muted responses and thus limit their potential to induce behavioral change.

## Introduction

Despite the known adverse effects of smoking on health (1), smoking remains the leading cause of preventable death in the world (2), with tobacco-related deaths projected to reach 8 million annually by the year 2030 (2). Since 2005, the World Health Organization Framework Convention on Tobacco Control has recommended including prominent, pictorial health warning labels (HWLs) on tobacco packaging. The goal is to effectively communicate the adverse effects of tobacco use to current and potential consumers of tobacco products, as well as to promote smoking cessation and prevent smoking uptake (2). In 2001, Canada became the first country in the world to implement pictorial HWLs. Since then, pictorial HWLs have been adopted and implemented in over 100 countries around the world. A key concern regarding HWLs concerns their “wear out” over time, i.e., the extent to which people adapt to the initially salient nature of the graphic component of the HWLs.

In 2009, the Family Smoking Prevention and Tobacco Control Act gave the U.S. Food and Drug Administration (FDA) the authority to select pictorial HWLs for cigarette packages in the United States with the explicit aim of increasing consumer understanding of smoking-related risks (3). In accordance with the law, the FDA proposed the content for nine new HWLs in 2012 (4), which address the topics of smoking-related health risks for smokers (e.g., lung disease, cancer, stroke), nonsmokers exposed to secondhand smoke, addiction, and the benefits of quitting (5). The specific HWL's selected by the FDA created considerable controversy. In January 2011, the Tobacco Control Legal Consortium (TCLC), the United States' legal network for tobacco control policy, submitted comments to the FDA advocating for more effective cigarette HWLs than those originally selected, suggesting that the proposed FDA HWLs were less graphic and less powerful than HWLs utilized in other countries (4). On the other hand, four of the five largest U.S. tobacco companies filed a lawsuit against the FDA and successfully blocked implementation of the selected HWLs, which they claimed violate their rights to free speech by compelling them to engage in a government campaign that is against their interests (6, 7). Nevertheless, the U.S. Supreme has upheld the FDA's mandate to inform consumers about the risks of smoking [11]. As a result, the FDA must undertake further research to support the selection of another set of pictorial HWLs for the US (7).

Observational and survey based studies show that pictorial HWLs are more effective than text-only HWLs in capturing attention (8–12), informing people about the risks associated with smoking (9, 13) and promoting smoking cessation (10, 11, 14).

More graphic pictorial HWLs appear to be more effective than other types of pictorial imagery. For example, Kees et al. (15) examined the effectiveness of *less* graphic, *moderately* graphic, and *highly* graphic pictorial HWLs on perceived fear, intentions to quit, attitude and message recall. Results indicated that the stronger the graphic pictorial warning was, the more it strengthened smokers' intentions to quit. Moreover, this effect was fully mediated by the fear that the image evoked. Other studies suggest that “graphic” depictions of disease are significantly more effective than images showing experiences of human “suffering” from the consequences of smoking or “symbolic” representations of risk (e.g., bomb to represent a pending heart attack) (16, 17).

To date, pictorial HWL effects have been evaluated primarily using self-reported responses, which may be subject to biases including telescoping, omission, and social desirability (18–24). One potential way to avoid such biases is to establish and record more objective biomarkers of HWL effectiveness. To this end, cognitive neuroscientists have begun to investigate the relationship between brain activity elicited during viewing of pictorial HWLs and its relationship to traditional measures of their effectiveness. Using this approach, researchers have identified a putative neural network responsible for processing pictorial HWLs which includes attentional networks (i.e., dorsolateral and dorsomedial prefrontal gyri; inferior parietal lobule), motor planning (supplementary motor area), temporal gyri (middle and superior aspects), limbic regions involved in memory (hippocampus) and affect (amygdala, insula), and visual processing regions (cuneus, precuneus, fusiform gyrus, and primary visual cortex) (25–28).

Across those broad networks, specific sites appear to be modulated by specific characteristics of HWLs that are known to be associated with cognitive and affective pathways that lead to behavioral change (15–17). For example, Newman-Norlund and colleagues recently reported that pictorial HWLs rated as more fear inducing elicited greater activation in the visual association cortex (BA 18) (25). And Wang et al. (27) found that HWLs that elicited a strong emotional response (ER) also elicited greater activity than low ER HWLs in the amygdala, hippocampus and prefrontal gyrus. Interestingly, neural responses to HWLs obtained using MRI appear to capture something that not reflected in traditional self-reported measures. Multiple studies show that MRI data, when added to self-reported data, significantly improve the accuracy of models designed to predict changes in smoking behavior (29–31).

Despite the nascent database of neuroimaging studies on mechanisms underlying response to HWL's, a number of important questions remain regarding the optimal design and deployment of graphic HWLs for tobacco products. One key question related to HWL effectiveness concerns the extent to which, and speed with which, smokers adapt or become accustomed to graphic HWLs. Based on the results of numerous brain imaging studies showing within-session adaptation to emotional images (32–36), it is reasonable to assume that the neural response to a given pictorial HWL will be maximal during the first exposure, and will decrease for repeated exposures. Understanding the nature of neural adaptation in the context of repeated exposure to pictorial HWLs, including which HWL characteristics produce the most durable effects, could help researchers and public policy experts maximize the effectiveness of these HWLs.

While one recent study found that current smokers' neural response to graphic images did decrease across multiple scanning sessions, this finding may have been influenced by changes in participant fatigue across the hour-long scanning protocol. Therefore, in order to directly examine the effects of repeated exposure to identical pictorial HWL stimuli, the current study examined adaptation over a shorter timeframe by means of a *repetition suppression fMRI* paradigm. Also known as *fMRI-adaptation*, this technique measures the extent to which BOLD signal is attenuated across rapid subsequent presentations of the same stimulus (37, 38) (as compared to rapid subsequent presentation of different stimuli).

The primary purpose of the current study was to evaluate neural response-suppression induced by the repeated sequential presentation of pictorial HWLs in current smokers. In order to address this question, we explored fMRI-adaptation effects in two distinct sets of graphic HWLs: foreign approved HWLs thought to be more vivid and emotionally powerful and proposed domestic HWLs, which are thought to be less vivid and emotionally powerful. First, we predicted that, within the putative HWL network, BOLD signal elicited by more graphic foreign HWLs would be greater than brain activity elicited by less graphic domestic HWLs. We predicted that current smokers exposed to repeated HWLs would show evidence of fMRI-adaptation effects (i.e., less robust activity relative to distinct HWLs), and that participants would show greater fMRI-adaptation to images thought to be less potent (domestic HWLs) than to images thought to be more potent (foreign HWLs. Again, we expected these differences to be localized to sites within the putative pictorial HWL network.

## Materials and methods

### Sample

Sixteen neurologically healthy smokers (11 males, 5 females) with no known neurological abnormalities or diseases and with normal or corrected-to-normal vision participated in this study. The age range was 18–36 years old (***M*** = 25) and all participants reported that they were daily smokers. All subjects self-reported that they were right-handed.

### Recruitment

Flyers to recruit participants were posted around the University campus and in local coffee shops, bars, restaurants, and other popular public venues. Phone screening for safety and eligibility criteria was conducted when participants contacted the study coordinator. Further safety screening was done through email, given volunteers passed the phone screening. A total of 17 individuals were screened for the study. A total of 17 individuals were screened for the study. One participant was excluded due to the presence of MRI incompatible IUD device. Participants were compensated with $50 for their time, which amounted to an hour and a half experimental session. All participants gave written informed consent and provided health information required to ensure MRI safety following a protocol approved by the local Institutional Review Board. Prior to scanning, each participant completed a standardized questionnaire about his or her smoking habits. This questionnaire revealed the following details regarding this group. The mean age when participants started to smoke was 17. On average, participants had smoked on 28.9 of the past 30 days and had smoked 12.2 cigarettes a day. One participant smoked a pipe as well as cigarettes.

### Protocol

**Figure 1:**
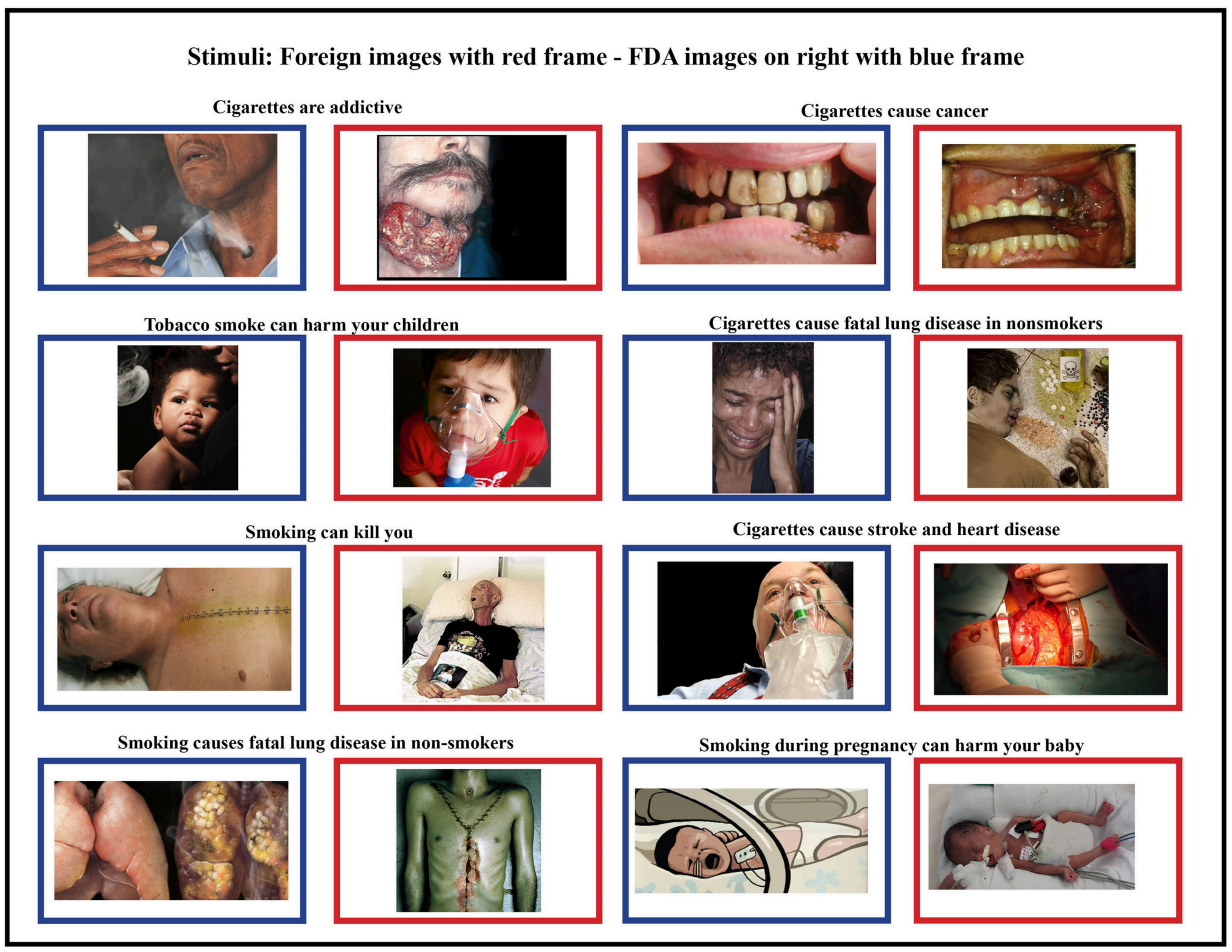
Foreign stimuli (blue frames) and corresponding domestic stimuli (red frames) were matched for content and health topic. Foreign HWLs are generally thought to be more graphic than domestic (FDA) HWLs (5, 17).

To examine brain activity, we asked each participant to view images presented on a computer screen. The images consisted of a subset of the proposed FDA images along with foreign HWLs images matched for health topic (Figure 1). Some of these images originally contained text, but were cropped to remove the text label as text in an individuals' native language might elicit reading regardless of task. All foreign images had been used on HWLs at time of the study. One of the nine FDA-proposed HWLs was excluded due to a non-removable text element. All data was collected between January 1, 2013 and February 1, 2012.

All images were proportionally interpolated to fit a 1024 × 768 resolution screen. We also created a set of scrambled images in order to estimate the amount of brain activity generated by higher-level visual recognition vs. low-level visual responses. These scrambled images were generated by Fourier transformation where the phase information was removed from the FDA and foreign HWL images using a MATLAB (The MathWorks, Inc., Natick, MA, USA) script (http://www.mccauslandcenter.sc.edu/CRNL/tools/spm8-scripts). Therefore, the scrambled images had similar low-level visual properties such as colors and spatial frequency but were not recognizable (Figure 1).

**Figure 2:**
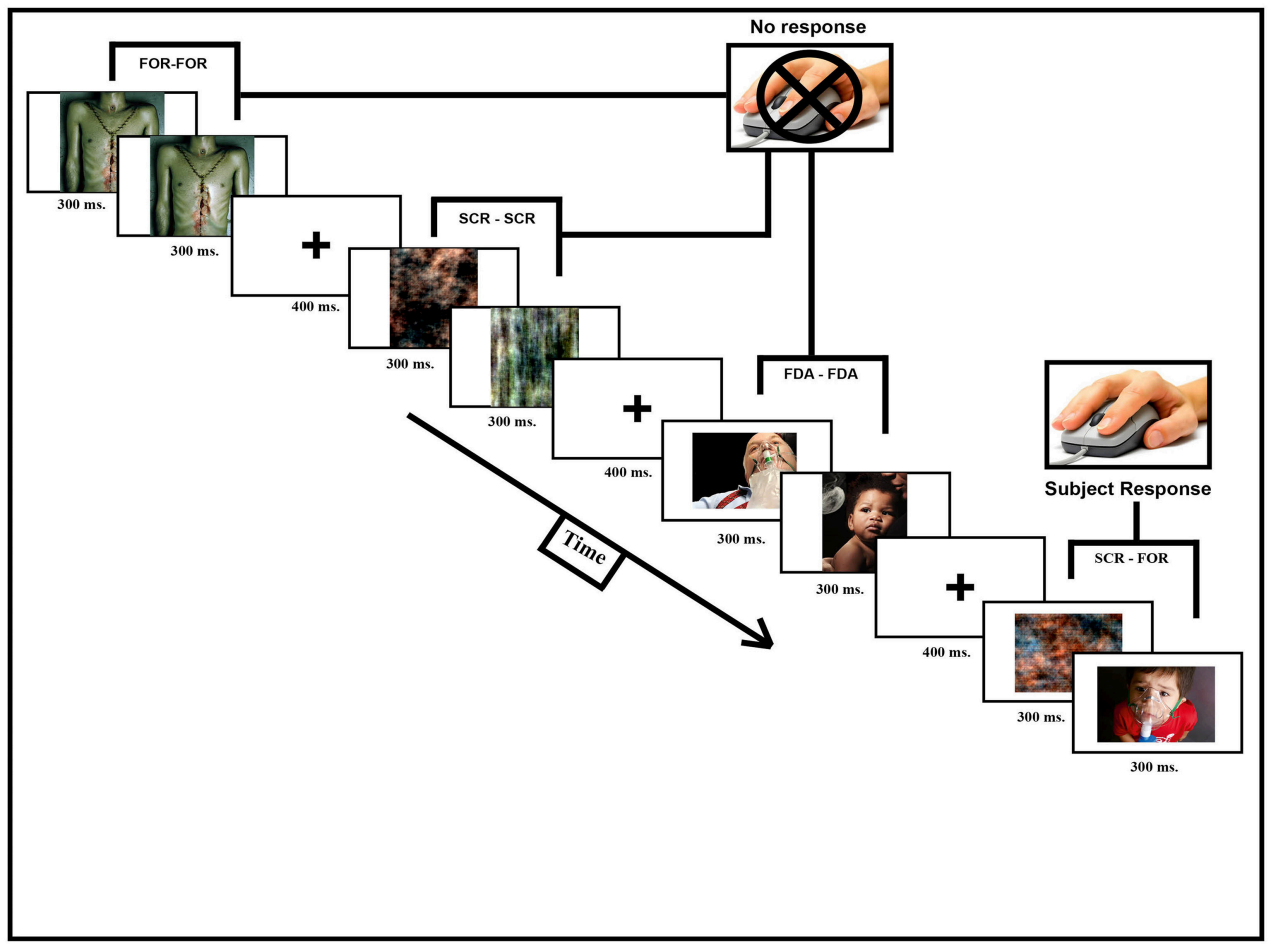
Participants were scanned while observing image pairs (600 ms, 300 ms per picture) separated by a short pause (400 ms). Image pairs could fall into one of seven different categories: HWL_ForCon_ (identical foreign HWLs), HWL_DomCon_ (identical domestic HWLs), HWL_ForIncon_ (different foreign HWLs), HWL_DomIncon_ (different domestic HWLs), SCR_Con_ (identical phase-scrambled images), SCR_Incon_ (different phase-scrambled images), or MisMatch (one phase-scrambled image and one HWL). The participant's task was to press a button whenever they observed a rare (11%) MisMatch trial.

During a single fMRI scanning session (consisting of a T1-weighted structural scan and 2, functional runs, each lasting 12 min and 13 s) participants observed a pairs of pictures shown in short succession followed by a pause of random duration. When HWLs were presented, half of the time they were shown in congruent trials that consisted of two identical images separated by a brief delay. These could be either two foreign HWLs (HWL_ForCon_) two domestic HWLs (HWL_DomCon_). The other half of HWL trials consisted of incongruent trials in which two different images were presented separated by a brief delay. Again, these could be either two foreign HWLs (HWL_ForIncon_) two domestic HWLs (HWL_DomIncon_). The presence of both congruent and incongruent trials allowed us to measure the response suppression effect (e.g., the reduction in response seen to repeated exposure vs. observing a novel stimulus). Additionally, we had trials in which congruent phase-scrambled images (SCR_Con_) or incongruent phase-scrambled images (SCR_Incon_) were shown. In a small percentage of trials (11%) the two images we presented were from different classes, i.e., one image was an HWL and the other was a scrambled image (MisMatch). We asked the participants to press a button whenever they observed a mismatched pair. This task was designed to ensure that the participant was observing the stimuli. This task was orthogonal to our experimental manipulations (i.e., the task was independent of whether the images were from the FDA or foreign HWLs) (Figure 2).

### Stimuli

Stimuli presentation and data collection was done using E-Prime software (Psychology Software Tools, Pittsburgh, PA). Each participant briefly practiced the task outside the scanner suite to familiarize them with the procedure and task. The participants were then tested inside the scanner where they observed a digital projector screen that was located outside the scanner via a mirror mounted on the scanners' head coil. Manual responses (i.e., button pressing) were made using a MRI compatible response glove. Each image was shown 13–16 times over the course of the experiment. The duration of presentation for each image was 300 ms, after showing a fixation cross for 400 ms. The inter-trial interval varied from 1,800 to 3,000 ms (Figure 2).

### Data acquisition

All MRI data were collected on a Siemens 3T Trio scanner at the McCausland Center for Brain Imaging, fitted with a 12-channel receiver head coil. During the first part of the scanning, the participants underwent a localizer and a structural scan. Next, the participants completed the two sessions of the tasks during continuous fMRI acquisition. Each session lasted 12 min and 13 s, with a T2^*^ echo planar imaging pulse sequence using the following parameters: repetition time, 2.130 ms; echo time, 35 ms; flip angle, 90°; 64 × 64 matrix; 192 × 192 mm field of view; 36 ascending 3.6-mm-thick slices with 20% slice gap, resulting in voxels with an effective distance of 3.25 × 3.25 × 3.6 mm between voxel centers with 344 volumes per session.

### fMRI data analysis

(http://www.fil.ion.ucl.ac.uk/). Data preprocessing was conducted in Statistical Parametric Mapping (SPM12) software and included motion correction, slice-time correction, spatial normalization, and spatial smoothing using an 8 mm full-width half-maximum Gaussian kernel. Voxelwise analysis was computed for 16 participants, excluding one subject due to numerous large head movements observed as more that 5 mm translation jumps between successive volumes (e.g., coughing). All subsequent statistical maps were thresholded at *p* < 0.001 adjusted, uncorrected, 10 voxel extent. Contrasts comparing conditions of interest were created at the first level, and then fed into 2nd level ANOVA's for hypothesis testing.

## Results

### Main effect of HWLs

**Figure 3:**
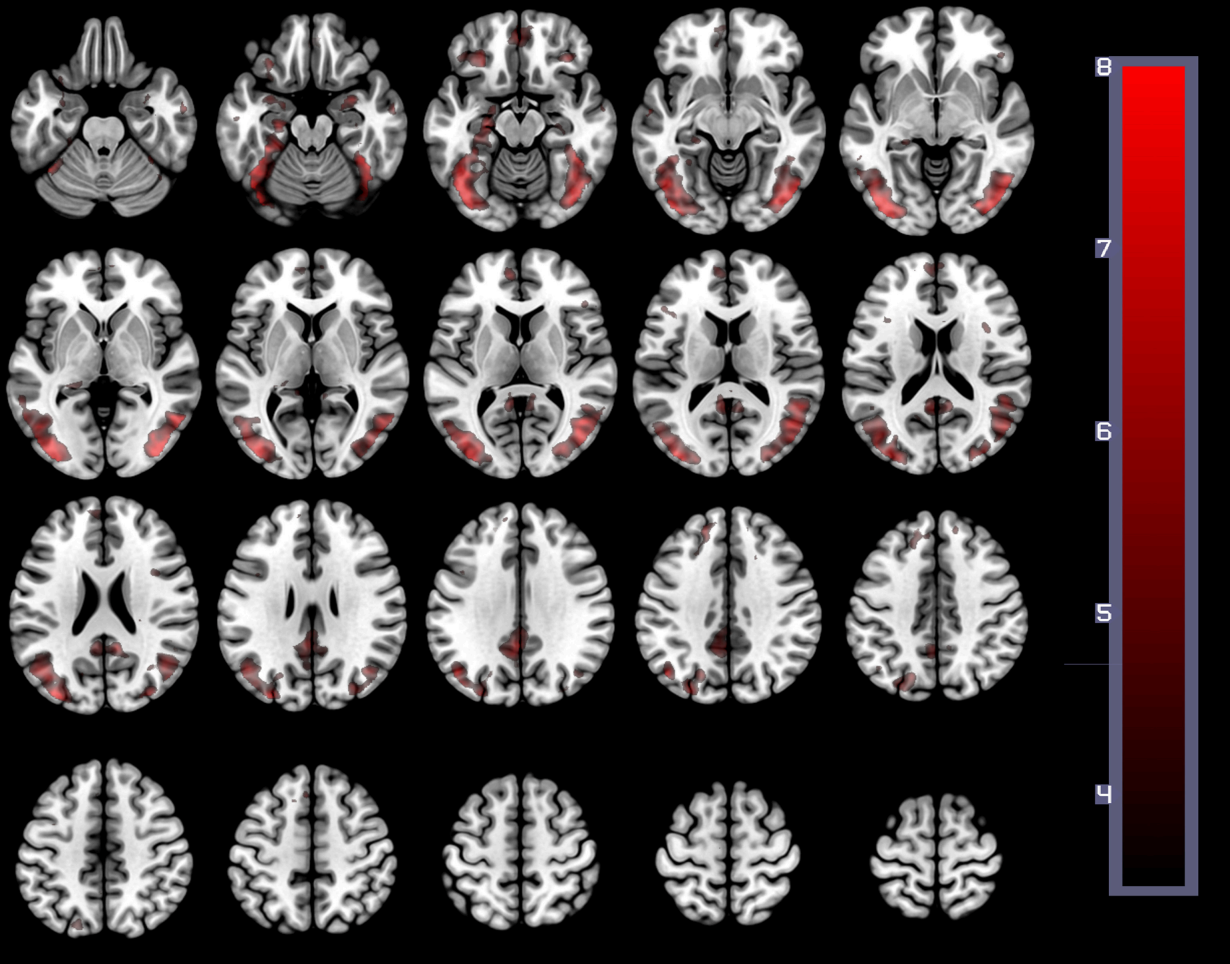
The main effect of HWLs as compared to scrambled images. HWLs elicited activation in the putative HWL network.

In order to establish the putative brain regions responding to all HWLs (foreign and domestic), we first computed a one-sample *T*-test using the contrast comparing pictorial HWLs (both foreign and domestic) to scrambled images ([HWL_ForCon_ + HWL_DomCon_ +HWL_ForIncon_ + HWL_DomIncon_]–[SCR_Con_ + SCR_Incon_]). As compared to control images, HWL's elicited increased BOLD response in regions previously identified as key nodes in the putative pictorial HWL network (25–28), including lateral prefrontal cortex(inferior, middle, medial, and superior frontal gyri), supplementary motor area, temporal gyrus (middle and superior aspects), inferior parietal lobe, limbic system (amygdala, hippocampus, insula), cuneus, precuneus, fusiform gyrus, and visual cortex. We also observed activation at sites in the cingulum, superior parietal lobule (SPL) (Table 1, Figure 3).

**Table 1:** Brain areas showing increased activation during viewing of health warning labels vs. scrambled images.

**HWLs** > **SCRAMBLED IMAGES**							
**LEFT**				**RIGHT**			
**Area**	**MNI**	**BA**	***Z***	**Area**	**MNI**	**BA**	***Z***
OCC_middle_	−38, −86, −6	27	12.35	OCC_middle_	42, −84, −2	28	15.8
Fusiform	−40, −58, −16	27	9.65	OCC_inferior_	44, −76, −14	28	12.93
Hippocampus	−26, −18, −18	29	8.73	MTG_posterior_	46, −62, 10	60	10.93
ANG	−46, −68, 34	63	8.32	MTG_posterior_	52, −60, −2	60	9.99
OCC_inferior_	−48, −74, −8	5	8.16	Fusiform	40, −60, 18	60	8.26
MTGpost	−40, −72, 20	63	8	STG_posterior_	50, −48, 16	70	8.25
IFG_orb_	−28, 34, −16	79	7.53	Precuneus	10, −50, 22	36	7.38
Precuneus	−4, −50, 16	49	6.69	Amygdala	30, 0, −20	54	6.71
Cingulum	−8, −54, 34	35	6.44	IFG_orb_	36, 32, −16	80	6.67
MTG_posterior_	−42, −66, 22	63	6.42	vMPFC/Rectus	0, 48, −16	21	6.15
SFG	−16, 40, 40	51	6.41	MTG_posterior_	42, −68, 28	64	5.97
MPFC	−8, 52, 8	19	6.25	MTGant	60, −6, −22	32	5.84
vMPFC/Rectus	0, 48, −16	21	6.15	IFG_oper_	36, 10, 20	82	5.03
SPL	−18, −72, 40	27	6.02	SFG	14, 42, 42	52	4.82
OCC_superior_	−26, −78, 42	27	5.39	Hippocampus	28, −20, −16	30	4.81
Amygdala	−30, 2, −20	53	5.28	IFG_tri_	48, 30, 8	76	4.74
IFG_tri_	−48, 26, 16	75	5.11				
IFG_oper_/PrG	−42, 8, 30	81	4.8				
SMA	−4, 24, 52	15	4.67				
MTG_anterior_	−48, 4, −30	29	4.26				

### Effect of foreign HWLs vs. US HWLs

A comparison of foreign and domestic HWLs ([HWL_ForCon_ + HWL_ForIncon_] + [HWL_DomCon_ + HWL_DomIncon_]) showed that foreign HWLs elicited significantly greater activation at sites in the bilateral precentral gyrus (PcG), bilateral middle occipital gyrus, right superior occipital gyrus, bilateral lingual gyrus, and right insula. (Table 2).

**Table 2:** Brain areas showing greater activity during observation of foreign, as opposed to domestic, health warning labels.

**FOREIGN** > **DOMESTIC**							
**LEFT**				**RIGHT**			
**Area**	**MNI**	**BA**	***Z***	**Area**	**MNI**	**BA**	***Z***
Lingual	−22, −88, −14	25	6.28	OCC_superior_	16, −100, 8	24	7.63
PcG	−34, 0, 46	11	5.29	Lingual	18, −90, −10	26	7.48
OCC_middle_	−26, −96, 8	23	5.05	Insula	40, 28, 2	80	5.2
				OCC_middle_	34, −86, 22	28	4.99
				PcG	24, −10, 48	12	4.94

### Main effects of repeated presentation

**Figure 4:**
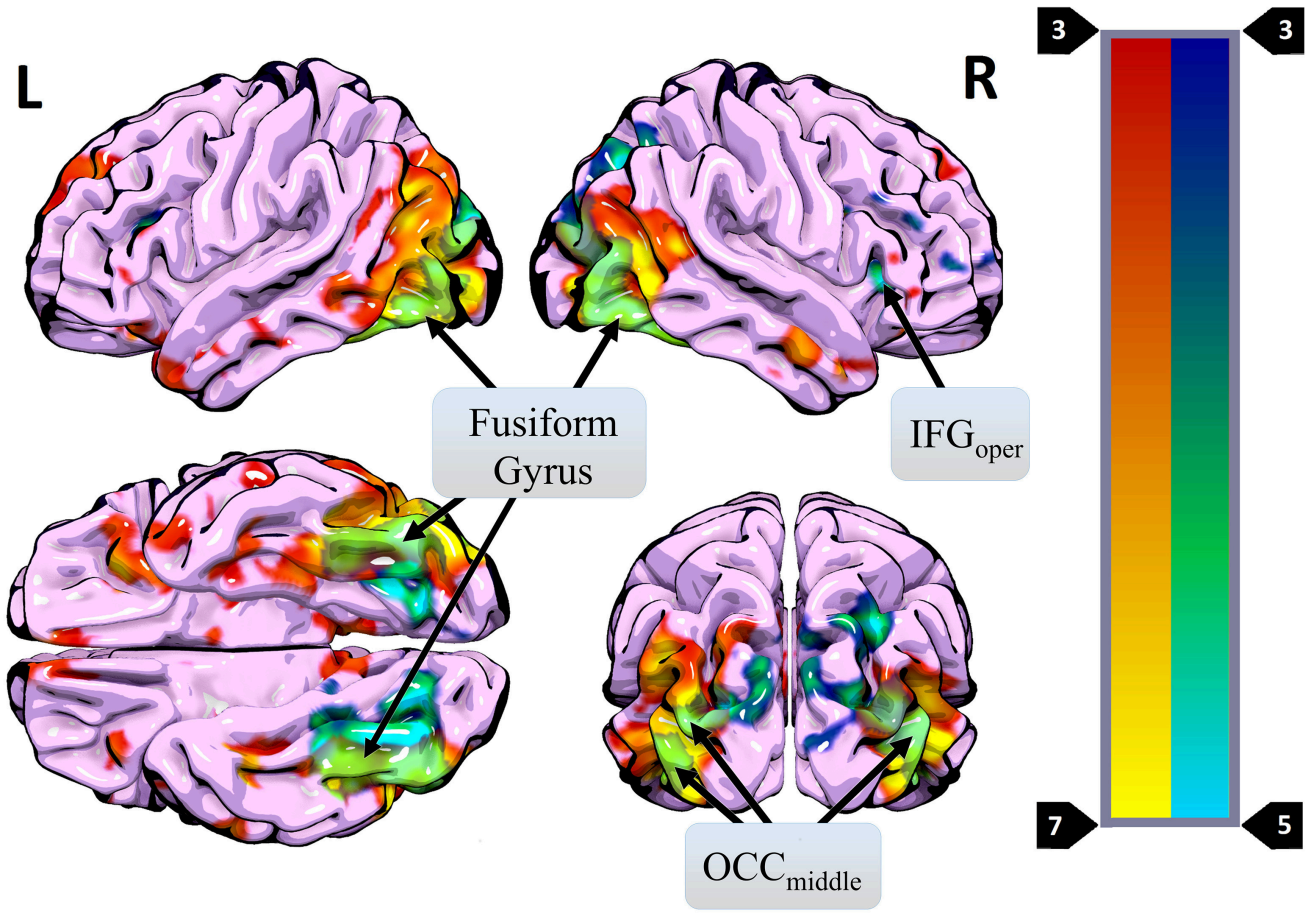
Regions where more activity was observed during presentation of different HWL images (vs. the same image two times in a row) (blue) overlaid on regions where more activity was observed for real images than phase-scrambled images (red). Areas of overlap are shown in light green. Regions active in both contrasts included the right IFG_oper_, bilateral OCC_middle_, and bilateral fusiform gyrus.

Repeated presentation of HWL (i.e., [HWL_ForIncon_ + HWL_DomIncon_]–[HWL_ForCon_ + HWL_DomCon_]) revealed main effect in the occipital cortex, fusiform gyrus, inferior frontal gyrus, middle frontal gyrus, angular gyrus, inferior temporal gyrus, lingual gyrus, cuneus, occipital cortex, inferior parietal lobule, and insula (Table 3, Figure 4). As an additional test, we compared the results of this contrast in the first and first and second fMRI runs. There was no significant difference in this contrast (i.e., [HWL_ForIncon_ + HWL_DomIncon_]–[HWL_ForCon_ + HWL_DomCon_]) when comparing fMRI run 1 and fMRI run 2 in the regions identified during the initial comparison.

**Table 3:** Brain areas showing greater activity following sequential presentation of two different health warning labels, as opposed to two identical health warning labels.

**HWL ADAPTATION EFFECTS**							
**LEFT**				**RIGHT**			
**Area**	**MNI**	**BA**	***Z***	**Area**	**MNI**	**BA**	***Z***
OCC_middle_	−26, −90, 20	27	8.58	Fusiform_anterior_	32, −50, −10	50	9.13
Fusiform_posterior_	−34, −72, −16	27	7.8	Fusiform_posterior_	32, −62, −12	28	8.89
Fusiform_anterior_	−30, −46, −20	59	6.04	OCC_inferior_	42, −66, −14	28	8.67
Lingual	−16, −74, −12	25	5.81	ITG	46, −50, −12	30	7.48
Cuneus	−10, −92, 28	27	5.06	OCC_superior_	26, −70, 44	14	7.36
OCC_superior_	−18, −86, 34	27	4.85	ANG	24, −58, 44	14	6.98
IFG_tri_	−40, 26, 20	81	4.66	Lingual Gyrus	18, −72, −10	26	6.35
Insula	−32, 22, 10	81	4.62	Insula	32, 26, −2	81	6.19
IPL	−24, −64, 40	65	4.34	OCC_middle_	26, −88, 22	28	5.99
				Cuneus	6, −78, 38	14	5.68
				Precuneus	6, −78, 38	14	5.68
				IFG_tri_	48, 32, 20	76	5.05
				IFG_oper_	36, 10, 20	44	5.03
				IFG_orb_	50, 18, 30	82	4.74
				Cingulate_middle_	4, 26, 40	52	4.68
				MFG	42, 46, 14	76	4.54
				IFG_tri_	50, 22, 2	76	4.3
				Cingulate_anterior_	4, 42, 32	52	4.13

### Effects of repeated presentation in foreign HWLs vs. US HWLs

Finally, we tested the prediction that participants would show greater fMRI adaptation effects (examined with the previous contrast) to images thought to be less potent (domestic HWLs) than to images thought to be more potent (foreign HWLs). Specifically, we compared the adaptation effect observed for domestic HWLs to the adaptation effect observed for foreign HWLs ([HWL_DomIncon_-HWL_DomCon_]–[HWL_ForIncon_-HWL_ForCon_]). No voxels survived this statistical comparison (*p* < 0.001 uncorrected, 10 voxel extent).

## Discussion

The present study used brain imaging to investigate cortical activity associated with passive viewing of pictorial HWLs. Our initial comparison of BOLD signal associated with HWLs and scrambled images demonstrated that, as expected, pictorial HWLs elicited more robust activation at sites associated with the processing of complex graphic visual images (Table 1, Figure 1), processing of complex visual stimuli (e.g., bilateral visual association cortex) (39–41), arousal/emotion (e.g., bilateral amygdala) (42–49), faces (e.g., fusiform gyrus) (50–53), and self-referential thoughts (vmPFC) (29, 30, 54, 55). These findings are in accordance with results from prior studies examining the neural correlates of viewing graphic pictorial HWLs (25–28) and add to the growing field of neuroimaging studies supporting the idea that pictorial HWLs exert their effects via activation of networks responsible for emotional and cognitive decision making (28).

A primary goal of the current experiment was to explore the functional correlates of neural adaptation associated with viewing HWLs. We expected participants to show evidence of fMRI-adaptation effects when presented with identical pictorial HWL stimuli (i.e., less robust activity relative to distinct HWLs), a phenomenon referred to as “repetition suppression” (RS) (56). As hypothesized, we observed widespread activation differences between trials in which repeated and novel HWLs were presented. Compared to the sequential presentation of identical HSWs, the sequential presentation of different HWLs elicited greater activation in a network that, to a large extent, mirrored the network already identified as being responsive to HWLs in general (vs. scrambled images) (Table 3). Sites within the putative HWL network that showed RS effects included the occipital cortex, fusiform gyrus, and right IFG pars opercularis (Figure 4).

The fusiform gyrus has long been known to play a critical role in face processing (50, 53). Moreover, this area is known to exhibit RS effects in response to face stimuli (38, 57–59). In the current experiment, 13 of 16 pictorial HWLs contained either full or partial (e.g., mouth, nose) images of human faces (Figure 1). One explanation for the widespread RS effects we observed is that they derived from RS effects originating in the fusiform gyrus. Prior work by Ishai et al. (32) suggests that RS associated decreases in fusiform activity can be associated with decreases at associated sites in the temporal sulci, IFG, insula, amygdala, and occipital gyri. All of these regions fall within the subset of areas shown to exhibit RS effects in the current experiment. The occipital cortex itself is known to show strong adaptation effects in response to repeated visual stimuli, regardless of emotional content. Vision specific RS occurs at stages as early as the retina (60) and as late as the visual cortex (61–63).

Interestingly, we did not observe the predicted interaction between the RS effect and image type. In other words, both foreign and domestic stimuli showed statistically similar RS effects. A study by Fischer and colleagues (64) that examined RS using repeated presentations of fearful and neutral stimuli failed to find an interaction between RS and stimulus type, suggesting similar neural attenuation rates to fearful and neutral stimuli. However, another study by Ishai et al. (32) that compared RS for fearful and neutral faces found that RS effects were indeed stronger for fearful, as compared to neutral faces. While our results appear to be consistent with this latter study, and therefore lend further credence to the idea that brain regions involved in processing faces habituate similarly regardless of stimulus type, it is possible that our choice of stimuli could explain our results. Specifically, foreign and domestic HWLs used in the current study, while certainly different, were much more similar to each other than the fearful and neutral faces used in these prior studies.

Based on the more graphic nature of the foreign, as compared to domestic HWLs used in the present study (5, 17), we predicted that the neural response to foreign HWLs would be greater than the response to their domestic counterparts. This was indeed the case, with foreign HWLs eliciting more robust activation at sites including the bilateral occipital gyrus and the left insular cortex (Table 2). Previous studies involving the pictorial HWLs have reported more robust activity for more graphic/arousing images at similar locations. For example, Newman-Norlund and colleagues reported that pictorial HWLs depicting graphic representations of the effects of smoking elicited a strong BOLD response in the visual association cortex relative to HWLs portraying suffering or symbolic effects of smoking (25). While we did not control for low-level features (color/luminance) or eye movements in the comparison between findings from studies suggests that the response of the visual cortex was unlikely due to low level characteristics of the HWL stimuli (25) or differential eye movements in the two conditions (41). Hyperactivation of the insular cortex in response to more graphic HWLs is also consistent with prior work comparing the neural correlates of more and less graphic HWLs. Historically, activation in the insula has been interpreted as representing disgust responses (65–68). Foreign HWLs such as those used in the current experiment are often described as being more graphic than domestic, FDA HWLs. This is certainly consistent with these data.

Independent of our failure to detect an interaction between RS and HWL type, our data demonstrate that the RS effect is robust for facial images embedded in emotionally charged graphic HWLs. This is clear evidence for some form of adaptation to repeated presentations of HWLs and provides strong justification for the consideration of RS effects with regards to the creation of effective HWLs. Currently, individual cigarette packages contain a single HWL which, presumably, the user is exposed to multiple times per day as they handle the product and remove cigarettes. Our data suggest that this approach to HWLs limits impact at the level of the brain. Given the known link between the neural response to pictorial HWLs and subsequent decision making/behavioral change (25, 27, 29–31), these results should be considered carefully as they have the potential to inform the design of better/more effective smoking-related HWLs. The development of advertising approaches capable of providing varied, as opposed to repetitive HWLs should be encouraged. For example, the use of lenticular printing to create images that change depending on viewing angle is one simple approach that may help overcome the RS effect.

This study has several important limitations. First, our sample size was relatively small (*N* = 16), and it may be that, with a larger sample we would find additional areas activated by observation of graphic HWLs. Our interpretation of the data is also limited by our failure to collect detailed self-reported ratings of individual HWLs from each individual, as well as our failure to collect information regarding participants' personality. Possession of such measures would allow the neuroimaging data to be examined in finer detail. The brain activation we report could have been influenced by participants smoking prior to the scanning session leading to alteration in cerebral blood flow in different brain areas (69, 70). Even though this potential artifact is worth mentioning, there is no reason to think that such a biological effect would only influence one stimulus type and not the other, or have an interaction with the effects of repeated vs. different images. It remains challenging to quantify cortical response to visual stimuli due to individual preferences and experiences. While an image of a woman holding a baby in the vicinity of white smoke (see FDA stimuli) may evoke neutral emotional response for someone that is not a parent it could very well trigger a negative reaction in a person who is a parent. Another possible limitation of this study is the lack of text accompanying the HWLs. Although we removed text from HWLs to eliminate factors involved with text processing and thereby focus solely on responses to picture stimuli, it is well known that textual elements give HWL imagery greater context (17, 71). Finally, our results may be difficult to generalize to the real world. While we demonstrate a significant RS effect associated with the presentation of two identical HSLs, smokers encounter the same HWL many more times in the course of a day. It is possible that the low-level RS we observed will not be directly related to an images ability to effectively elicit cortical responses over a period of months. Future behavioral and neuroimaging studies should examine the RS effect following three, four, or even more repetitions. And longitudinal studies might examine the impact of these images after realistic delays (time between purchasing cigarette packages at the store, time between cigarettes) on the effects of HWLs.

The current study utilized fMRI to examine neural adaptation to graphic HWLs in a population of current adult smokers. Presentation of graphic HWLs elicited strong activation in the putative HWL network. This network responded most robustly to trials in which two different HWLs were presented, while the response was muted when two identical HWLs were presented. While this RS effect was not different for foreign and domestic HWLs, suggesting that the more graphic nature of the former did not impact RS, it may be that emotional content of the pictures were not sufficiently different to support such an effect. When considered with prior studies indicating a strong relationship between the amplitude of neural responses to HWLs and subsequent behavioral change, these preliminary data suggest that researchers focusing on the development of more effective smoking cessation programs should consider ways to minimize repetition of HWLs and maximize variety in the presentation of graphic images portraying the negative health consequences of smoking. Given the exploratory nature of this study, more extensive fMRI research is needed on different types of HWLs, with different study designs, including prior exposure to HWLs as in the real world, in order to better understand their effects on neural activity and behavior. Furthermore, there is a need to explore potential effectiveness of HWLs by cross-validating smokers' self-reported responses, including a focus on regions like the medial prefrontal cortex, an area which has previously been associated with behavior change (30, 72).

## Ethics statement

This study was carried out in accordance with the recommendations of the University of South Carolina IRB. The protocol was approved by the University of South Carolina IRB. All subjects gave written informed consent in accordance with the Declaration of Helsinki.

## Author contributions

JF and CR designed the study and wrote the protocol. JF and RN-N undertook the statistical analysis. JF, CR, and JT wrote the first draft of the manuscript. All authors contributed to and approved the final manuscript.

### Conflict of interest statement

The authors declare that the research was conducted in the absence of any commercial or financial relationships that could be construed as a potential conflict of interest.
